# Insights from letter position dyslexia on morphological decomposition in reading

**DOI:** 10.3389/fnhum.2015.00143

**Published:** 2015-07-03

**Authors:** Naama Friedmann, Aviah Gvion, Roni Nisim

**Affiliations:** ^1^Language and Brain Lab, Tel Aviv UniversityTel Aviv, Israel; ^2^Reuth Medical and Rehabilitation CenterTel Aviv, Israel; ^3^Communication Sciences and Disorders, Ono Academic CollegeTel Aviv, Israel

**Keywords:** morphological decomposition, Hebrew, letter position, inflection, derivation, letter position dyslexia, acquired dyslexia, developmental dyslexia

## Abstract

We explored morphological decomposition in reading, the locus in the reading process in which it takes place and its nature, comparing different types of morphemes. We assessed these questions through the analysis of letter position errors in readers with letter position dyslexia (LPD). LPD is a selective impairment to letter position encoding in the early stage of word reading, which results in letter migrations (such as reading “cloud” for “could”). We used the fact that migrations in LPD occur mainly in word-interior letters, whereas exterior letters rarely migrate. The rationale was that if morphological decomposition occurs prior to letter position encoding and strips off affixes, word-interior letters adjacent to an affix (e.g., signs-signs) would become exterior following affix-stripping and hence exhibit fewer migrations. We tested 11 Hebrew readers with developmental LPD and 1 with acquired LPD in 6 experiments of reading aloud, lexical decision, and comprehension, at the single word and sentence levels (compared with 25 age-matched control participants). The LPD participants read a total of 12,496 migratable words. We examined migrations next to inflectional, derivational, or bound function morphemes compared with migrations of exterior letters. The results were that root letters adjacent to inflectional and derivational morphemes were treated like middle letters, and migrated frequently, whereas root letters adjacent to bound function morphemes patterned with exterior letters, and almost never migrated. Given that LPD is a pre-lexical deficit, these results indicate that morphological decomposition takes place in an early, pre-lexical stage. The finding that morphologically complex nonwords showed the same patterns indicates that this decomposition is structurally, rather than lexically, driven. We suggest that letter position encoding takes place before morphological analysis, but in some cases, as with bound function morphemes, the complex word is re-analyzed as two separate words. In this reanalysis, letter positions in each constituent word are encoded separately, and hence the exterior letters of the root are treated as exterior and do not migrate.

## Introduction

Major questions in the study of morphological processing are whether and when morphological decomposition takes place during reading. Since the seminal work of Taft and Forster (1975), many researchers assume that words are represented in a decomposed form in the orthographic input lexicon. If this is so, then in order to identify a word in the lexicon, morphological decomposition is required. Debates remain as to whether this decomposition is obligatory or whether words can still be accessed as wholes: Taft and Forster (1975) supported an obligatory decomposition account (see also Taft, 2004; Taft and Ardasinski, 2006; Rastle and Davis, 2008), whereas other models advocated a dual-access view whereby morphologically complex words can also be stored as wholes in the lexicon and decomposition occurs only in certain conditions (e.g., Schreuder and Baayen, 1995; Baayen et al., 1997; Diependaele et al., 2009, 2013). Additional work has revolved around the question of the nature of the morphological decomposition: whether it is guided by purely structural, morphological-orthographic considerations (Longtin et al., 2003; Rastle et al., 2004; Longtin and Meunier, 2005; Rastle and Davis, 2008; Beyersmann et al., 2011; Crepaldi et al., 2014) or rather consults the lexicon or lexical-semantics (Giraudo and Grainger, 2000, 2001).

In the current study, we ask when and how this morphological decomposition takes place using letter position encoding. Specifically, we ask about the interaction between letter position encoding and morphological decomposition, and their relative order. Until now, studies that asked questions about morphological decomposition by using letter transpositions examined priming in normal readers. The basic idea of many of these studies was to compare the priming of primes created from existing words by transposition within the stem to primes created by transposition across morpheme boundaries. A difference in the priming effect of the two conditions would indicate that morphological decomposition occurs early. These studies yielded inconsistent results (Christianson et al., 2005; Duñabeitia et al., 2007; Grainger and Ziegler, 2011; Rueckl and Rimzhim, 2011; Masserang and Pollatsek, 2012; See Sanchez-Gutierrez and Rastle, 2013; Taft and Nillsen, 2013, and Amenta and Crepaldi, 2012, for review and discussion). A different way to look at morphological decomposition through transpositions was created by Beyersmann et al. (2011; see also Beyersmann et al., 2013). Their idea was to examine priming from a morphologically complex nonword created from a transposed stem and a suffix to the stem. Their findings, indicating priming in such stimuli, point to morphological decomposition. Finally, in a recent study, Taft and Nillsen (2013), who also used priming in normal reading, took advantage of the fact that primes in which the exterior letters transposed provide a smaller priming effect primes with middle transposition. They compared transpositions at the exterior letters of the stem (which would be exterior letters following decomposition) to transpositions in the middle of the stem: comparing, for example, *disrpove*, and *disporve*, respectively. Their results were that even when the prime was a nonword, when it could be decomposed to a lexical stem and existing affix (e.g., *unprove*), it primed a word with the same stem, indicating early morphological decomposition. No difference was found in the priming of exterior and middle transpositions, which the authors explained by saying that the reduced effect of initial letters is purely perceptual and hence this was not observed once the initial letters of the stem were not perceptually initial.

In the current study we looked at morphological decomposition through letter position from a novel perspective: that of the reading pattern of individuals with letter position dyslexia (LPD), a dyslexia that specifically affects letter position encoding. The rationale is the following: LPD affects an early, pre-lexical stage of orthographic-visual analysis (for these model components c.f., Ellis and Young, 1996; Coltheart et al., 2001; Jackson and Coltheart, 2001; Friedmann and Coltheart, in press). Therefore, whether or not LPD is affected by the morphological structure of the target word can inform us about morphological processing taking place in this early stage.

Previous studies have already examined the interaction of morphological decomposition and peripheral dyslexias—dyslexias in the pre-lexical orthographic-visual analysis stage. Reznick and Friedmann (2009) tested the effect of morphology on the reading of 7 Hebrew readers who had word-based neglect dyslexia (neglexia) following stroke. Neglexia is a reading deficit in which letters on one side of the word are neglected, causing substitutions, omissions and additions of letters on the neglected side (Caramazza and Hillis, 1990; Ellis and Young, 1996; Haywood and Coltheart, 2001; Vallar et al., 2010). Readers with left neglexia may read *stop*, *unclear*, and *cars*, as “top,” “clear” and “bars,” respectively. Readers with right neglexia would read *boot*, *liver*, and *corner* as “book,” “live/lived” and “corn.” Reznick and Friedmann found that the reading of the neglexic patients was affected by the morphological structure of the target words: affixes were neglected significantly more than root letters. This pattern was especially evident in letter omission errors: whereas affixes on the neglected side were often omitted, root letters were never omitted. This effect was purely structural and was not affected by lexical properties of the root and the target word. The interpretation was that morphological decomposition affects reading already in the orthographic-visual analysis stage, and without feedback from the lexical stages: it requires three root letters, and does not stop shifting attention to the neglected side until three root letters are found.

A similar effect of morphology on peripheral dyslexia was found in the reading errors of 10 individuals with developmental attentional dyslexia (Friedmann et al., 2010b). The typical error in attentional dyslexia is the migration of letters between neighboring words. Friedmann et al. found that these errors occurred more often in affix morphemes than in the root.

Neglexia and attentional dyslexia both stem from a pre-lexical deficit at the orthographic-visual analyzer: neglexia affects attention shift to the neglected side of the word and attentional dyslexia affects binding of letters to words. Therefore, the findings of both studies serve as an additional evidence that morphological decomposition indeed occurs very early in the course of word reading, before lexical access.

The current study assessed a different function of the orthographic-visual analysis stage, which possibly functions at an earlier stage than letter-to-word binding^1^: that of letter position encoding. We asked whether the morphological structure of the target words affects letter position errors in LPD, to find out whether letter position encoding precedes or follows morphological decomposition. We further asked whether all types of morphemes behave similarly or whether they exhibit different patterns with respect to decomposition. LPD is characterized by letter position errors in reading (e.g., trail→ trial, smile→ slime, cloud→ could) that occur mainly in middle letters (Friedmann and Gvion, 2001, 2005; Friedmann and Rahamim, 2007, 2014; Friedmann et al., 2010a; Friedmann and Haddad-Hanna, 2012, 2014; Kohnen et al., 2012; Kezilas et al., 2014). This dyslexia results from a selective impairment in letter position encoding in the early, pre-lexical stages of visual analysis of the written word.

We used the fact that individuals with LPD make transpositions in middle letters but almost never in the first or final letters. The idea was that if the morphologically-complex word is decomposed to its morphemes prior to the stage at which letter position errors occur, then the exterior letter of the base morpheme that is adjacent to an affix and therefore appears as a middle letter in the complex word, may become an exterior letter when stripped of the affix. For example, in a word like *signs*, the “n” is a middle letter, but if the plural affix −s is stripped off the base before the stage in which letter position errors occur, then the “n” becomes exterior and hence would not migrate.

Namely, if both conditions are fulfilled: morphological decomposition occurs before letter position encoding, and this decomposition actually creates two separate morphemes, then letter position errors are not expected to occur in base letters on the edge of an affix (or are expected to occur in a rate similar to that of exterior letters). If, however, letter position encoding (and hence, letter position errors) occurs prior to the early morphological decomposition, then at the level in which letter position errors occur, the first letter of the second morpheme is still in middle position, and would have a similar fate to other middle letters. In this case, it will show the same transposition rate as middle letters. To examine this question and to compare various types of morphemes, we used Hebrew, a morphologically-rich language.

### Morphology in hebrew

Hebrew is a Semitic language, read from right to left. It is an alphabetic script in which not all vowels are represented orthographically. Hebrew words are built from a 3-letter root and a derivational template and/or inflectional morphology. Verbs, nouns, adjectives, and prepositions can inflect for gender, number, and possessor/genitive. Verbs also inflect for tense and person. Derivational templates exist for verbs, nouns, and adjectives. The nominal template for nouns and adjectives is called “mishkal” and the verbal template for verbs is called “binyan” (Arad, 2005; Arad and Shlonsky, 2008). Inflectional and derivational morphemes may be vowels or consonants. The morphological structure of Hebrew words was consistently shown to affect word reading. For example, in a line of priming studies and oral reading in rapid serial visual presentation, Frost et al. (1997, 2000a,b), and Velan and Frost (2007, 2009, 2011) showed that Hebrew words prime visual recognition of other words that share their roots (more than other orthographically similar primes).

As shown in Appendix A Table A1, the morphological inflections and the derivational templates may appear before, in the middle, or at the end of the word. Many of them occur in more than one position in the word. One can think of the morphemes in Hebrew as a template consisting of consonants and vowels, with three empty slots for consonants, in which the root letters are inserted. All 22 Hebrew letters can function as root letters, 12 letters can also be part of inflectional or derivational affix. Some letters can serve as inflectional or derivational affixes only in the beginning of the word, but not in its end (e.g., 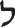), whereas other letters can appear as affixes before, within, and after the root (e.g., 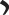), or both before and after the root (e.g., 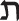,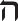). Some morphemes are single letters, whereas others are two letters. There is another type of morpheme in Hebrew, which we term “bound function morpheme.” These are 7 function words that appear in English as separate words (*the*, *that*, *and*, *in*, *to*, *as*, *from*). In Hebrew they appear as a single letter (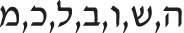, respectively) that is bound to the beginning of the word, and appears as part of the word (theword, andappears). Bound function morphemes always precede the word^2^. We compared in this study inflectional, derivational, and bound function morphemes, assessing whether they are stripped off the words early enough so as to make the adjacent root letters behave like exterior letters.

## Participants and background tests

### Background description of the 12 participants with LPD

The participants were 11 individuals with developmental LPD and one woman with acquired LPD following brain damage. Galia, the participant with acquired dyslexia, was a 54 years old woman. She was a teacher and a PhD student with 20 years of education. She had a sudden onset of seizures with herpes encephalitis 13 months before our testing. CT demonstrated a small hypodense area in the right temporal lobe. Her reading was impaired, showing clear and selective LPD. Her speech and naming abilities were normal. Her writing was impaired, with mild graphemic buffer dysgraphia. She participated only in Experiment 1, in which the participants read aloud 500 migratable words. The background details of the developmental LPD participants, who were all school students, 5 females and 6 males, are summarized in Table 1.

**Table 1:** **Background description of the participants with developmental LPD**.

**Participant**	**Age**	**Gender**	**Grade**
YO	18;1	M	12
OR	13;11	M	8
BR	13;7	M	7
MR	12;2	F	6
EL	11;6	M	6
AD	11;1	M	6
TL	11;5	M	6
SK	11;7	F	5
AF	11;5	F	5
YV	11;0	F	5
TA	10;5	F	5

### Testing to establish LPD and for inclusion in the study

Each of the participants with LPD was selected to participate in this study on the basis of migration errors within words in reading aloud and in silent reading, alongside intact word production. This screening testing included two tasks of reading aloud: the TILTAN screening test of oral reading of 136 single words of various types, and a test of oral reading of 232 migratable words. To establish that the migrations that the participants made in reading indeed resulted from a deficit in letter position encoding and not in the speech production stages, we also used tasks of reading without oral production: a test of migratable word comprehension, and tests of oral production without reading: picture naming and migratable word repetition. We only included participants who made migrations in reading aloud and in comprehension and who had no migrations in oral word production.

#### Screening tests

The *TILTAN reading screening test* (Friedmann and Gvion, 2003) includes 136 single Hebrew words of various types that were constructed so that they are sensitive to various types of dyslexia: Most importantly for our study, 65 of the words in the test are sensitive to detect LPD as these words are migratable words—words for which a transposition of middle letters can create another existing word. All the words in the test are sensitive to left neglect dyslexia at the word level, as all the words in the list are such that when read with a neglect error on the left side (omission or substitution of letters), another existing word can be created (such as *snow*, which can be read as “know” or “now” following a left letter substitution or omission, respectively); 104 of the words are sensitive to right neglect, as neglect errors on their right side create other existing words. The test also includes words for identifying surface dyslexia^3^: potentiophones and words that are parallel to irregular words in English; abstract words, function words, and morphologically complex words, for identifying deep dyslexia (and phonological output buffer dyslexia); words with many orthographic neighbors for identifying visual dyslexia; and words for which migrations, substitutions, omissions, or additions of a vowel letter create other existing words for identifying vowel dyslexia (Khentov-Kraus and Friedmann, 2011).

For individuals who made significantly more migration errors than controls, without other dyslexias, who were therefore suspected to have LPD, we further administered an additional reading aloud test of 232 migratable words.

The *232 migratable words oral reading test* includes 232 Hebrew words in which migration of middle letters creates another existing word (such as cloud-could, parties-pirates, casual-causal). The 232 migratable words had 4–7 letters (*M* = 4.9, *SD* = 0.9). In 87 of these words a middle migration that involves a vowel letter and a consonant letter creates another existing words, and in 163 words a middle migration that involves only consonant letters creates another word.

To establish that the impairment is at the early stage of orthographic-visual analysis rather than in the output stages, we also tested reading comprehension of migratable words, picture naming, and the repetition of 20 migratable words. The rationale was that if the deficit is at the orthographic-visual analysis stage, not only reading aloud but also comprehension of migratable words would be impaired and indicate transpositions of middle letters, but picture naming and repetition should not be affected. An output deficit should show the opposite pattern, with good comprehension of written migratable words when no reading aloud is required, and poor oral production in picture naming and repetition.

*Reading comprehension* of migratable words was tested using 50 triads of written words. Each triad included a target migratable word, a word that is semantically related to it and a word that is semantically associated to the transposition counterpart of the target word. The participant was requested to choose the word that was related to the target word. For example, the target word 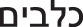, dogs, in which a transposition creates the word 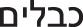, cables, appeared with the words *animals* and *television*.

*Naming* was tested using a picture naming task of 100 color object pictures (*SHEMESH*, Biran and Friedmann, 2004); *repetition* was tested using a task of repetition of 20 migratable words.

#### Results in the screening tests of LPD participants included in the study

We selected only participants who had significantly more letter migration errors on the three tasks of migratable word reading than age-matched skilled readers (TILTAN norms, Friedmann and Gvion, 2003), using Crawford and Garthwaite's (2002) *t*-test for the comparison between an individual and a control group, and who performed normally and migrations-free in picture naming and repetition.

Table 2 summarizes the participants' reading performance– number of errors of each type—in the TILTAN reading screening test. Table 3 summarizes their performance in oral reading of the 232 migratable word test and their performance in reading comprehension of the 50 migratable words.

**Table 2:** **Number of errors of the various types in the TILTAN oral reading screening test**.

**LPD participants**	**Transposition errors**	**Surface-dyslexia-like errors**	**Vowel letter addition**	**Vowel letter omission**	**Vowel letter substitution**	**Consonant letteraddition**	**Consonant letter omission**	**Consonant letter substitution**	**Semantic substitutions**
Developmental									
YO	7^*^	6^*^	1	0	0	0	1	0	0
OR	22^*^	4	4^*^	0	0	1	5^*^	1	0
BR	18^*^	5	1	0	0	1	1	0	0
MR	11^*^	9^*^	3	2^*^	0	0	0	3^*^	0
EL	13^*^	8^*^	1	2^*^	0	0	3^*^	2	0
AD	6^*^	3	0	0	0	0	0	0	0
TL	20^*^	5^*^	4^*^	2^*^	1	5^*^	6^*^	5^*^	0
SK	17^*^	5^*^	3	1	0	0	1	0	0
AF	21^*^	2	3	2^*^	0	1	2^*^	0	0
YV	13^*^	4	1	1	0	0	0	1	0
TA	14^*^	12^*^	3	2^*^	1	2^*^	5^*^	1	0
Acquired GALIA	15^*^	4	0	0	0	0	0	0	0
Average	14.8	5.6	2.0	1.0	0.2	0.8	2.0	1.1	0

* *Significantly more errors than age-matched control group (p < 0.05)*.

**Table 3:** **Percentage of migrations in oral reading of 232 migratable words and errors in a task of comprehension of 50 written migratable words**.

**LPD participants**	**Migrations in oral reading of 232 migratable words %**	**Migrations in comprehension of migratable words %**
**DEVELOPMENTAL**		
YO	13^*^	12^*^
OR	27^*^	50^*^
BR	10^*^	34^*^
MR	7^*^	37^*^
EL	13^*^	22^*^
AD	9^*^	19^*^
TL	15^*^	25^*^
SK	27^*^	34^*^
AF	24^*^	56^*^
YV	12^*^	44^*^
TA	27^*^	50^*^
Acquired GALIA	22^*^	59^*^
Average	17.2	36.8

* *Significantly more errors than age-matched control group (p < 0.05)*.

In reading aloud, as can be seen in Tables 2 and 3, the prominent error of all the participants was letter migrations within words, and each of them made significantly more migration errors compared to age-matched controls, whereas other types of reading errors were relatively few^4^.

The comprehension of migratable words, which involved only silent reading, also indicated that the participants had LPD, as each of them made significantly more errors than the controls in this test.

Unlike their impaired oral and silent reading, characterized by migration errors, the participants' naming and migratable word repetition was normal, and none of the participants made migration errors in naming or in repetition. Table 4 summarizes their performance in the picture naming and repetition tasks.

**Table 4:** **Naming and word repetition performance of the participants with developmental LPD**.

**Participant**	**Picture naming (%correct)**	**Repetition of migratable words (%correct)**
YO	98	100
OR	98	100
BR	100	100
MR	96	100
EL	99	100
AD	100	100
TL	99	100
SK	99	100
AF	97	100
YV	99	100
TA	98	100

This pattern of results shows that indeed the source of the migration errors of the 12 participants lies in the encoding of letter position in the orthographic visual analyzer.

### Control group

The control group included 25 age-matched skilled readers without any reading impairments, as tested by the *TILTAN* reading screening test. They were 9 female and 16 male. Ten of them were age-matched to the 5–6th grade participants with LPD (mean age = 11.6, *SD* = 0.5); Ten were age-matched to the 7–8th grade participants with LPD (five matched to the 7th grade participant and five to the 8th grade participant, mean age = 13.8, *SD* = 0.9); and five were 12th grades, age-matched to the older individual with LPD (mean age = 18.4, *SD* = 0.4). In all the data tables below, YO was compared to the 12th grade group, OR and BR to the 7–8th grade group, and the rest were compared to the 5–6th grade control group. These control participants were tested in all the reading tests that were administered to the LPD participants, described in the following sections.

## General method

The experimental study of morphology in LPD included six experiments that tested reading, lexical decision, and comprehension of migratable words in two levels: single words, and sentences that include migratable words.

### Procedure

During the testing sessions, every response that differed from the target was transcribed by the experimenter, and words read correctly were scored with a plus sign. All the sessions were audio-recorded and two judges listened to the recordings after the sessions, and the transcription from the session was checked and corrected or completed using the recordings.

The words and sentences in the various experiments were presented to each participant over the desk, printed on a white page. In the oral reading tasks, the participant was requested to read aloud as accurately as possible; in the lexical decision and comprehension tasks the participant was requested to perform the task without reading the words aloud. No time limit was imposed during testing, and no response-contingent feedback was given by the experimenter, only general encouragement. The participants were told that whenever they needed a break they can stop the session or take a break. Each participant was tested individually in a quiet room in two to three sessions of 1–2 h. The Ethics Committees of Tel Aviv University and the Ministry of Education approved the experimental protocol.

### Data analysis

The results were analyzed on the group level as well as for each individual participant. We compared the performance at the group level between two conditions using *t*-test for correlated samples (after we established that the data of the LPD participants on the inflectional, derivational, bound, and exterior conditions did not depart from normality, as the skewness and kurtosis of each of them did not significantly differ from 0).

At the individual level, performance in different structures was compared using Chi square test. To compare the performance of each experimental participant to her/his age-matched control group, we used Crawford and Howell's (1998; Crawford and Garthwaite, 2002) *t*-test. An alpha level of 0.05 was used.

## General materials: stimuli structure

Across all 6 experiments, we examined three types of morphemes: Inflectional, derivational, and bound function morphemes (conditions 1–3 below). In all cases, we examined the rate of transpositions of root letters AB that were adjacent to the tested morpheme, compared with a control condition in which the root letters AB were exterior^5^ (example 4, with and without an affix in the irrelevant side).

In 1–4 below, ABX represent the three consonant root letters. In all the target words, the two letters to be migrated, A and B, were always adjacent to each other, and the transposition of the letters AB created an existing word with the sequence BA in the relevant side.

**Table:** 

Condition 1: Inflectional morphology	
a. in the beginning:	[inflectional morpheme]**AB**X
b. in the end:	X**AB** [inflectional morpheme]

**Table:** 

Condition 2: Derivational morphology	
a. in the beginning:	[derivational morpheme]**AB**X
b. in the end:	X**AB**[derivational morpheme]

**Table:** 

Condition 3: Bound function morpheme	
in the beginning:	[bound function morpheme]**AB**X

**Table:** 

Condition 4: Exterior letter migration, with no morpheme on the relevant side	
a. in the beginning:	**AB**X(possibly a morpheme here)
b. in the end:	(possibly a morpheme here)X**AB**

We followed several procedures and principles when creating the list of words of the various types: we used the same root for the various conditions, in most cases (72% of the roots) the same root was used in all 4 conditions or in 3 conditions, except when the root does not naturally appear with some of the morpheme types. That way, in many cases it was exactly the same root and the same two letters that migrated in the compared conditions. For example, the 3-letter root 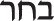, bxr (and here x is the IPA transcription of the velar fricative consonant represented by the letter 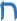, not a variable), which has a transposition counterpart 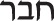, xbr, appeared in the inflectional condition with an inflectional prefix (and affix) as 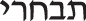 (tbxri, you-will-choose), with the transposition counterpart 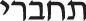 (txbri, you-will-connect); in the derivational condition, with a derivational prefix, as 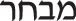 (mbxr, selection), with the transposition counterpart 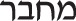 (mxbr, connector or connects); in the bound function morpheme condition as 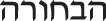 (hbxorh, the-girl), with the transposition counterpart 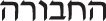 (hxborh, the-group or the-bound); and in the exterior transposition condition as 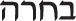 (bxrh, selected-fem), with the transposition counterpart 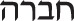 (xbrh, girlfriend or company). In each of these four conditions the relevant transposition involves the first two root letters.

The derivational morphemes were morphemes of verbal and nominal templates, the inflectional morphemes were morphemes of person, gender, number, tense, and possessive pronoun suffixes.

The Hebrew bound function morphemes always appear before the root, and so they did in the stimuli. We used the 7 bound function morphemes, in a way that they always formed a syntactically licit combination with the word they were bound to (e.g., the determiner “the” and the preposition “in” were always added to a noun or an adjective but not to a verb).

In the bare root control condition, we used the 3-letter root itself, when it was an existing word. In the morphologically complex exterior letter migration condition, we used the root and an additional affix that appeared on the side opposite to the expected migration—if the expected exterior migration was on the beginning, in letters 1 and 2 of the root, the affix was added at the end of the word, and if the expected migration was at the end, the affix was added in the beginning of the word, before the root. The morphologically complex control condition also included vowel letters inside the words, but not between the migrating letters, which were always adjacent. The longer control stimuli were used so that exterior letter migration would be tested in words of the same length as the words in the morphological conditions.

In Hebrew, five letters have different forms in middle and final position, so in order to avoid the effect of letter form on position encoding (see Friedmann and Gvion, 2005), these letters did not appear in any of the morphological conditions when the migration of the second and third root letters was tested, either as the second or as the third root letter.

Morphologically complex words were classified to the various conditions according to the type of morphemes that were adjacent to the site of expected migration. Namely, if a word started with an inflectional prefix and ended with a derivational suffix, it was considered part of the inflectional condition if the relevant migration was adjacent to the prefix (root letters 1 and 2), and part of the derivational condition if the relevant migration was adjacent to the suffix (root letters 2 and 3). In some of the word lists there were few words that had a potential for both migrations of the first and second root letters and the second and the third root letters. In these cases, we included these items in the totals of both conditions, and analyzed the errors according to the errors each participant made. The words of the various conditions were presented in a semi-random order, making sure that no more than two words of the same condition appeared consecutively, and that words of the same root (and even words with the same root letters in a different order) never appeared consecutively.

To assess the effect of the morphological structure of the word on the rate of migrations, we compared the rate of migrations of the two root letters (AB) in each of the three types of morphemes to the exterior migration^6^, and between the various morpheme types. Error scoring referred only to transposition errors and ignored surface dyslexia-like errors, so that words that were read with surface dyslexia-like errors but without transposition errors were counted as correct response.

We used three types of tasks: oral reading, lexical decision, and written word comprehension. Because we had initially thought that some morphological analyses may occur only within a sentence context, we examined each task both on the single word level and in the sentence level, with a total of six experiments. (As you will see below, this worry was unwarranted, as morphological decomposition occurred even at the single word level). The group with LPD read a total of 8679 morphologically complex migratable words in the 6 experiments. Together with the initial lists of migratable words that each participant read in the screening stage, each participant read 1136 migratable words, so our results are based on a total of 12,496 migratable words that the LPD group read.

## Experiment 1: oral reading of single morphologically-complex words

### Method and material

Each participant was presented with a list of 500 words and was requested to read them aloud as accurately as possible. The word list included:

**116** words with initial **bound function morphemes** (Table 5, condition 1);
**104** words with **inflectional morphology** adjacent to the migrating letters: 52 in the beginning, 55 in the end (three of the 104 words included “relevant affixes” on both sides: in these words, both a migration of the root letters adjacent to an inflectional prefix and a migration near an inflectional suffix created an existing word) (Table 5, conditions 2a and 2b);
**109** words with **derivational morphology** adjacent to the migrating letters: 56 in the beginning, 57 in the end (4 of the words included both a derivational prefix and a derivational suffix, each of which was adjacent to migrating root letters) (Table 5, conditions 3a and 3b).

**Table 5:** **Types of words that were included in the various conditions**.

	**Condition**	**Hebrew target → transposition**	**Transliteration target → transposition**	**Phon. Transcription target → transposition**	**Translation target → transposition**
1	Initial bound function morpheme	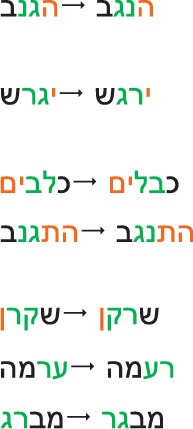	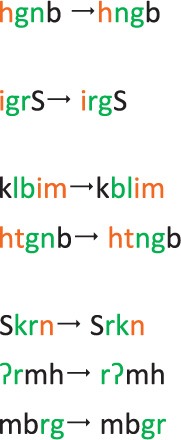	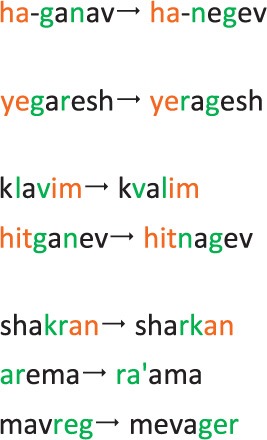	the-thief → the-Negev, southern Israeli region
2a	Initial inflectional morpheme				drive-out-3sg-mas-future → excite-3sg-mas-future
2b	Final inflectional morpheme				dogs → cables
3a	Initial derivational morpheme				sneaked-3sg-mas-refl → dried-3sg-mas-refl
3b	Final derivational morpheme				liar → Guinea pig
4a	Control initial exterior letter				pile → mane
4b	Control final exterior letter				screwdriver → make-older

The control items were **98** monomorphemic and **115** morphologically complex words in which a migration of two adjacent exterior letters created another word. The monomorphemic words included 39 words in which a transposition of the first two letters creates an existing word, 26 words in which a transposition of the last two letters creates an existing word, and 30 in which both the first-second transposition and final-penultimate transpositions create existing words (42 of the morphologically complex words served both in the exterior migration condition and in one of the morpheme conditions, when one side of the word allowed for an exterior migration and the other for migration adjacent to a morpheme); the morphologically complex words were matched in length to the words in the experimental conditions. In the morphologically complex control words the affixes were always on the other side of the words than the expected transposition. They included 56 words with a suffix, in which a transposition of first two letters creates an existing word, and 59 words with a prefix, where a transposition of last two letters creates an existing word (see Table 5, conditions 4a and 4b). The words in the different conditions did not differ in frequency [*F*_(3, 499)_ = 1.56, *p* = 0.20].

### Results

#### Participants with developmental LPD

The results, summarized in Table 6, indicated that the rate of transposition errors crucially depended upon whether the transposing letters were adjacent to an inflectional or derivational morpheme, or to a bound function morpheme: Whereas transpositions were abundant for all participants near inflectional and derivational morphemes, they were very scarce near bound function morphemes. Transpositions near bound function morphemes occurred in a low rate that was similar to the rate of exterior letter migrations. This pattern held at the group level and for each of the individual participants. At the group level, transposition errors occurred significantly more often in letters adjacent to inflectional [*t*_(10)_ = 7.22, *p* < 0.001, *d* = 2.6] and derivational [*t*_(10)_ = 8.09, *p* < 0.001, *d* = 2.9] morphemes than in letters adjacent to bound function morphemes, with no difference between the inflectional and derivational conditions. Similarly, transposition errors occurred significantly more often in letters adjacent to inflectional [*t*_(10)_ = 6.90, *p* < 0.0001, *d* = 2.7] and derivational [*t*_(10)_ = 7.78, *p* < 0.0001, *d* = 3.0] morphemes than in the exterior letters (with no difference in the rate of exterior letter migrations between the two control conditions—the bare root condition of 3-letter words and the longer morphologically-complex control condition—t_(100)_ = 1.52, *p* = 0.13). Importantly, the rate of transpositions edging a bound function morpheme did not differ from the rate of transpositions of exterior letters.

**Table 6:** **Percentage migrations in oral reading of single words according to the type of morpheme adjacent to the migration site**.

**LPD Participant**	**Inflection**	**Derivation**	**Bound function morpheme**	**Exterior root letters**
YO	15^*^	24^*^	3^*^	1^*^
OR	19^*^	21^*^	1	3^*^
BR	13^*^	11^*^	1	1^*^
MR	7^*^	12^*^	3^*^	3^*^
EL	12^*^	13^*^	3^*^	5^*^
AD	11^*^	17^*^	1	0
TL	15^*^	8^*^	4^*^	3^*^
SK	27^*^	31^*^	9^*^	4^*^
AF	16^*^	18^*^	7^*^	4^*^
YV	9^*^	15^*^	2^*^	2^*^
TA	24^*^	21^*^	3^*^	8^*^
Galia (Acquired)	27^*^	27^*^	1	4^*^
LPD Average (*SD*)	16.2 (6.9)	18.2 (6.9)	3.1 (2.7)	3.2 (2.1)
**CONTROL GROUPS**				
12th graders	1.2 (0.4)	1.7 (2.0)	0.3 (0.5)	0.2 (0.2)
7–8th graders	0.9 (1.1)	1.6 (1.5)	0.2 (0.4)	0.05 (0.1)
5–6 graders	1.1 (0.04)	4.0 (2.3)	0.3 (0.2)	0.1 (0.3)

* *Significantly more transposition errors compared with age-matched control group (p < 0.05)*.

The same tendency was found for each of the individual participants. All individuals made significantly (*p* ≤ 0.01) fewer transpositions near bound function morphemes than near inflectional and derivational morphemes (except for MR's inflectional vs. bound comparison, which was in the same direction but not significant). Similarly, each participant made significantly fewer transpositions in exterior letters than near inflectional (*p* ≤ 0.001) (apart from MR) and derivational (*p* < 0.05) morphemes. The number of transpositions near inflectional and derivational morphemes did not differ for any individual participant, neither did the bound and exterior letter conditions (*p* > 0.05).

Another finding sheds light on the early morphological analysis that occurred in the reading of our LPD participants: In total, across all 11 developmental LPD participants in reading all 500 migratable words, there were 58 exterior letter migrations in which two consonant letters transposed (1% of the words they read). None of these involved a root letter transposing with a letter that belonged to the bound function morpheme (or, in fact, any non-root morpheme). Even if we take only words in which an exterior transposition creates an existing word, there were 34 words (a total of 408 target words for all LPD participants) that started with a bound function morpheme, in which a transposition of the letter of the function morpheme and the first letter of the root could create an existing word (e.g., OBDK, vebadak, and-checked, that could create BODK, bodek, checks). However, this error occurred only once—only one participant made one such exterior migration across a morpheme boundary. This supports the conclusion that letter position errors occurred later than the morphological decomposition of the function morpheme from the word to which it was bound.

Additional analyses that explored decompositions and letter position errors in words in which the same letter can function in two different morphological roles are reported in Appendix B).

#### The woman with acquired LPD

Similarly to the participants with developmental LPD, Galia (see Table 6) also made transposition errors but mainly adjacent to inflectional (27.3% errors) and derivational (26.9% errors) morphemes. She made only few transpositions near bound function morphemes (3 errors) and in exterior letters (5 errors). Her transpositions near bound function morphemes were significantly fewer than near inflectional and derivational morphemes (χ^2^ = 18.63, *p* < 0.0001; χ^2^ = 17.6 *p* < 0.0001, respectively). Similarly, her transpositions in exterior letters were significantly fewer than her transpositions near inflectional or derivational morphemes (χ^2^ = 59.55, *p* < 0.0001; χ^2^ = 55.65, *p* < 0.0001, respectively), with no significant difference between the inflectional and derivational conditions (χ^2^ = 0.005, *p* = 0.94). Importantly, she made similar rates of migrations in exterior letters and near bound function morphemes, χ^2^ = 0.55, *p* = 0.46. The two control exterior-migration conditions (bare root and longer words) did not differ, χ^2^ = 0.39, *p* = 0.53.

### Interim summary: transpositions and morphological structure in reading aloud of single words

Both the developmental and the acquired LPD participants made significantly more transposition errors near inflectional and derivational morphemes than near bound function morphemes, and their transpositions near bound function morphemes were as scarce as exterior transpositions. No differences were found between the rate of transpositions near inflectional and derivational morphemes. These results indicate that some form of very early morphological decomposition applies to bound function morphemes, at the same time or before letter position encoding takes place. As a result of this early analysis, the bound function morpheme is stripped off the base word, so that the letters at the edge of the word that are adjacent to the bound function morpheme are treated as exterior letters, and hence, very few transpositions occur in them.

## Experiment 2: oral reading of migratable words in sentences

Experiment 1 indicated that when words are presented in isolation, there is an effect of early morphological decomposition on migrations in oral reading. Experiment 2 tested the effect of morphology on migrations in oral reading of migratable words in sentences.

### Materials and methods

The target words were migratable words in which a transposition of root letters could occur adjacent to inflectional, derivational, or bound function morphemes. The test included 30 sentences: the **inflectional condition** included 8 sentences with a word that allowed for a lexical transposition next to an inflectional morpheme (example 5); the **derivational condition** included 7 sentences with a word that allowed for a lexical transposition next to a derivational morpheme (example 6); and the **bound function word condition** included 15 sentences with a word that allowed for a lexical transposition next to a bound function morpheme (example 7, one of the items in the bound function morpheme condition was later excluded from the analysis because many of the participants read the target word with an irrelevant vowel error). Examples (5)-(7) demonstrate sentences of the three conditions, followed by the result of the expected transposition in parentheses.

(5) **Inflectional condition:**
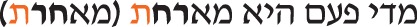
: She occasionally hosts (is-late)
(6) **Derivational condition:**
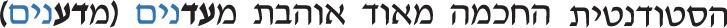
: The smart female-student likes very much dairy-puddings (scientists)
(7) **Bound function morpheme condition:**
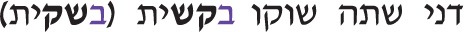
: Danny drank chocolate-milk in-straw (in-plastic-bag)

The migratable words in the different conditions did not differ in frequency [*F*_(2, 27)_ = 0.75, *p* = 0.50]. The sentences of the various conditions were presented in random order. We constructed the sentences in a way that both the target word and the word that results from the transposition would be syntactically, semantically, and pragmatically plausible in the sentence.

Error analysis focused solely on migrations in the relevant target words. We removed from the analyses 7 sentences in which the participant made an irrelevant (non-transposition) error on the target word.

### Results

The results of the reading aloud of migratable word within sentences are summarized in Table 7. Similarly to Experiment 1, in sentence context as well, the LPD participants made significantly fewer transpositions near bound function morphemes than near inflectional morphemes, *t*_(10)_ = 7.11, *p* < 0.0001, *d* = 2.0, and significantly fewer transpositions near bound function morphemes than near derivational morphemes, *t*_(10)_ = 2.71, *p* = 0.02, *d* = 1.1. There was no difference between the migration error rates in the inflectional and the derivational conditions. Each of the participants showed the same pattern, with no differences between the inflectional and derivational conditions (*p* > 0.05) but with more transpositions adjacent to inflectional and derivational morphemes than adjacent to bound function morphemes (*p* < 0.05). Due to the relatively small number of items, this difference reached significance at the individual level only for four LPD participants.

**Table 7:** **Migration errors in oral reading of migratable words in sentences (Percentage migrations out of the words presented in the relevant condition)**.

**Developmental LPD participants**	**Transposition near inflection**	**Transposition near derivation**	**Transposition near inflection+derivation**	**Transposition near bound morpheme**
YO	38^*^	14	27^*^	21^*^
OR	50^*^	14	33^*^	21^*^
BR	14	29^*^	21^*^	0
MR	38^*^	14	27^*^	0
EL^a^	13	0	7	0
AD	25^*^	14	20^*^	7
TL	25^*^	33^*^	29^*^	14^*^
SK	25^*^	57^*^	40^*^	14^*^
AF	25^*^	14	20^*^	14^*^
YV	25^*^	29^*^	27^*^	8
TA	38^*^	43^*^	40^*^	17^*^
LPD Average (*SD*)	28.7 (11.2)	23.7 (16.3)	26.5 (9.5)	10.5 (8.1)
**CONTROL GROUPS**				
12th graders	2.5 (5.6)	5.7 (7.8)	4.0 (3.7)	2.9 (3.9)
7–8th graders	7.5 (8.7)	5.7 (7.4)	6.7 (6.3)	0.7 (2.3)
5–6th graders	3.8 (6.0)	12.9 (8.1)	8.0 (4.2)	1.4 (4.5)

* *Significantly more errors than age-matched control group, p < 0.05*.

a *Indeed, in this task EL performed not differently from the matched controls, but he performed significantly worse than matched controls in most of the other tasks. In general, all participants performed below the control participants in all or most of the tasks reported here*.

## Experiment 3: lexical decision of single words

After we established the clear effect of the morphological structure of the target word on the rate of transpositions on the edge of the root in oral reading, we moved to assess whether the same effect is present also in reading tasks that do not involve reading aloud. Experiments 3 and 4 tested migrations in a lexical decision task at the single word and sentence level respectively.

### Materials and methods

The stimuli list for lexical decision included 105 items: 59 pseudowords and 46 non-migratable real words. The pseudowords included: 20 pseudowords derived from real words by transpositions of the root letters next to an inflectional morpheme (Table 8, examples 1a and 1b); 20 pseudowords derived from real words by transpositions of the root letters next to a derivational morpheme (Table 8, examples 2a and 2b); and 19 pseudowords derived from a transposition of exterior letters (Table 8, examples 3a and 3b). (This task did not include bound function morphemes because we realized it would be unnatural to request the participant to circle words, when a word with a bound function morpheme, parallel to, for example, “that-morning” could be considered as two words). The side of the transposition in the pseudowords—left or right, was controlled—there were half expected migrations on the right and half on the left in each condition (9 and 10 on the left and right in the exterior condition respectively). The items were presented in a random order on a paper and the participants were requested to circle only the real words, without reading aloud.

**Table 8:** **Types of pseudowords that were included in the lexical decision task**.

	**Condition**	**Pseudoword presented**	**Word created by transposition**	**Transliteration pseudoword**	**Transliteration word**	**Translation word**
1a	Initial inflectional morpheme	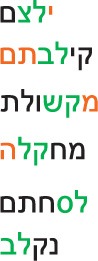	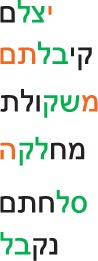	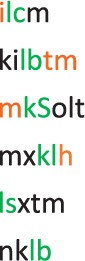	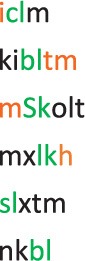	photograph-3sg-mas-fut
1b	Final inflectional morpheme					receive-past-2pl-mas
2a	Initial derivational morpheme					weight
2b	Final derivational morpheme					department
3a	Control initial exterior letter					forgave-2nd-pl
3b	Control final exterior letter					receive-1st-pl-fut

### Results

The results of the lexical decision task, summarized in Table 9, exposed the same pattern: there were significantly more transpositions next to inflectional and derivational morphemes than exterior transpositions [*t*_(10)_ = 4.59, *p* = 0.001, *d* = 0.9; *t*_(10)_ = 5.96, *p* = 0.0001, *d* = 1.4, for inflectional and derivational morphemes, respectively]. Inflectional and derivational morphemes did not differ significantly.

**Table 9:** **Percentage errors in lexical decision of migratable nonwords at the word level**.

**LPD Participants**	**Transposition near inflection**	**Transposition near derivation**	**Transposition of exterior root letters**	**Transposition in real words**
YO	10^*^	30^*^	11	0
OR	35^*^	25^*^	11^*^	7^*^
BR	40^*^	45^*^	5	0
MR	10^*^	5	0	11^*^
EL	15^*^	40^*^	0	4^*^
AD	15^*^	40^*^	0	4^*^
TL	15^*^	20^*^	16^*^	0
SK	70^*^	75^*^	32^*^	0
AF	65^*^	75^*^	47^*^	0
YV	25^*^	35^*^	11^*^	0
TA	60^*^	55^*^	32^*^	0
LPD Average (*SD*)	32.7 (23.0)	40.5 (21.6)	15.0 (15.6)	2.4 (3.7)
**CONTROL GROUPS**				
12th graders	3.0 (2.7)	7.0 (7.6)	4.2 (4.4)	1.6 (1.7)
7–8th graders	3.0 (3.5)	3.5 (5.3)	2.6 (2.8)	1.2 (2.3)
5–6th graders	0.5 (1.6)	4.0 (5.2)	1.1 (2.2)	1 (3.3)

* *Significantly more errors than age-matched control group (p < 0.05)*.

Each of the individual participants showed this pattern of more errors on pseudowords that involved transposition next to an inflectional / derivational morpheme than on pseudowords derived by transpositions of exterior letters. This difference was significant or approached significance (*p* ≤ 0.08) for six of the participants. The inflectional and derivational conditions did not differ significantly, neither on the group level, nor for any individual participant.

Thus, like in the oral reading Experiments (1 and 2), individual and group level analyses indicate that lexical decision is also vulnerable to migrations when the pseudoword is derived by transposing letters next to inflectional and derivational morphemes, whereas exterior transpositions are rare. This suggests that letters on the edge of the root, adjacent to an inflectional/derivational morpheme, are considered middle letters by the position encoding procedure.

## Experiment 4: lexical decision of migratable nonwords in sentences

To further test the effect of sentential context on migratability of letters near morphemes, we administered a lexical decision test using migratable words incorporated in sentences. Presenting the transposition errors within sentences allowed us to also include transpositions next to bound function morphemes, which we could not use in the single word lexical decision task.

### Materials and methods

A total of 64 sentences were presented to each participant: 8 sentences with a pseudoword that was formed by transposing the root letters near an inflectional morpheme (example 8); 7 sentences with a pseudoword formed by transposing the root letters near a derivational morpheme (example 9), and 15 sentences with a pseudoword formed by transposing the root letters near a bound function morpheme (example 10). The 9 control sentences included a pseudoword formed from a real word by the substitution of a single letter (example 11), and could not form any existing word following transposition. Twenty five length-matched sentences written correctly were presented as fillers.

The participants were requested to read each sentence silently and to judge whether the words in the sentence are written correctly or not.

(8) Migration near inflection: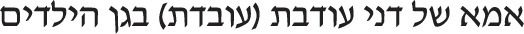  Ɂo**dv**t (Ɂo**vd**t) - o**d**e**v**et (o**v**e**d**et, work-3rd-sg-fem-present)
  Danny's mother pseudoword (works) in the kindergarten
(9) Migration near derivation: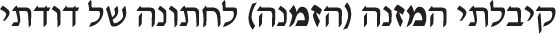  h**mz**nh (h**zm**nh) – ha**mz**ana (ha**zm**ana, invitation)
  I received pseudoword (an invitation) to my aunt's wedding
(10) Migration near a bound function morpheme: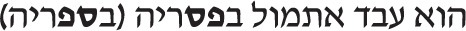  b**fs**rih (b**sf**rih**) –** ba**f**i**s**riya (ba**s**i**f**riya, in-the-library)
  He worked yesterday pseudoword (in the library)
(11) Letter substitution control: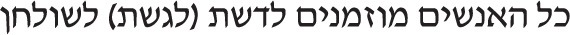  ldSt (l**g**St) - la**d**eshet (la**g**eshet, to approach)
  All people are invited pseudoword (to approach) the table

### Results

The lexical decision task in which the transposed pseudowords were incorporated into sentences yielded similar results to Experiments 1–3 (see Table 10). As Table 10 demonstrates, whereas the LPD participants were able to detect transpositions on the edge of a bound function morpheme quite well, at a level that was not significantly different from the letter substitution control condition, they were less likely to identify transpositions near an inflectional or a derivational morpheme. They detected significantly fewer errors next to an inflectional morpheme than next to a bound function morpheme *t*_(10)_ = 6.11, *p* < 0.001, *d* = 1.6, and fewer errors next to a derivational morpheme than next to a bound function morpheme, *t*_(10)_ = 2.49, *p* = 0.03, *d* = 0.9. The inflectional and derivational morpheme conditions did not differ significantly from each other. The inflectional and derivational conditions were both significantly poorer than the control letter substitution condition, *t*_(10)_ = 9.38, *p* < 0.0001, *d* = 2.9; *t*_(10)_ = 3.63, *p* = 0.005, *d* = 1.5, respectively.

**Table 10:** **Percentage errors on lexical decision of migratable nonwords within sentences**.

**LPD Participant**	**Transposition near inflection**	**Transposition near derivation**	**Transposition near bound morpheme**	**Letter substitution (control)**	**Correct sentences**
YO	63^*^	86^*^	50^*^	0	0
OR	38^*^	29^*^	29^*^	0	4^*^
BR	25^*^	0	14^*^	0	0
MR	38^*^	14	21^*^	11^*^	4^*^
EL	25^*^	14	0	0	0
AD	25^*^	29	0	0	12^*^
TL	38^*^	57^*^	7	0	20^*^
SK	50^*^	14	21^*^	11^*^	0
AF	50^*^	57^*^	29^*^	0	4^*^
YV	25^*^	14	0	0	0
TA	63^*^	71^*^	7	33^*^	0
LPD Average (*SD*)	39.8 (14.7)	35.1 (28.1)	16.2 (15.7)	5.1 (10.3)	4.0 (6.4)
**CONTROL GROUPS**					
12th graders	2.5 (5.3)	2.9 (4.5)	1.4 (3)	0 (0)	0.8 (1.7)
7–8th graders	1.3 (4.0)	1.4 (4.5)	0.7 (2.3)	0 (0)	0.4 (1.3)
5–6th graders	3.8 (8.4)	7.1 (12.1)	1.4 (3.0)	0 (0)	0.8 (1.7)

* *Significantly more errors than age-matched control group (p < 0.05)*.

At the individual participant level the pattern was similar, all but one participant performed more poorly on the inflectional and derivational conditions (combined) compared with the bound function morpheme condition, a difference that was significant for 3 of the participants. There were no differences between the inflectional and derivational conditions for any of the LPD participants.

## Experiment 5: comprehension of single written migratable words

Another task we used to examine whether the effect of different morphemes on migrations occurred also in silent reading was a comprehension task. Again, we tested word comprehension in a single word task (Experiment 5) and in words incorporated in sentences (Experiment 6).

### Materials and methods

We tested the comprehension of 60 migratable words using a word association task. Each migratable word was presented as part of a triad that included, in addition to the target migratable word, a pair of words, one was semantically related to the target word, the other was semantically related to a transposition error in the target word (examples 12–15). The participants were requested to choose the word that is semantically related to the target word, without reading the target word aloud. Again, the target migratable words in the test were of the four types: 15 words with potential of lexical transposition near an inflectional morpheme (12); 15 words with a potential of lexical transposition near a derivational morpheme (13); 15 words with a potential of lexical transposition error near a bound function morpheme (14), and 15 words with a potential of lexical transposition that involved exterior letters (15). In this task too, the inflectional, derivational, and exterior conditions included both words in which the transposition could occur on the left or adjacent to a relevant morpheme on the left of the word, and words in which the transposition was expected on the right. The target words of the various conditions did not differ in frequency [*F*_(3, 56)_ = 1.47, *p* = 0.23].

(12) migration near inflection:
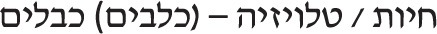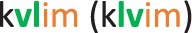 - kvalim (klavim)
  cables (dogs) - television / animals
(13) migration near derivation:
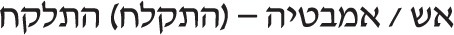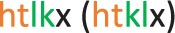 - hitlakeax (hitkaleax)
  caught a fire (took a shower) – fire/bath
(14) migration near bound function morpheme:
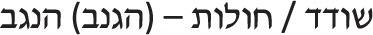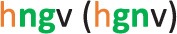 - hanegev (haganav)
  the Negev, a southern Israeli desert zone (the thief) – sands / robber
(15) exterior migration:
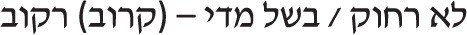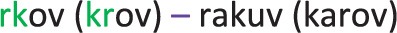  rotten (near) – too ripe / not far

### Results

The performance of each participant in each condition is presented in Table 11. In general, the performance of the LPD participants in this task was relatively good compared to the other tasks, possibly because this was the only task that explicitly presented two options, which may have caused more deliberate attempt to read the words letter-by letter to avoid transpositions. Still, in this task too, the participants performed poorer on triads that involved transposition near inflection and derivation compared to triads that involved transposition near bound function morphemes or transposition of exterior letters.

**Table 11:** **Percentage errors in the comprehension of single migratable words**.

**LPD Participants**	**Transposition near inflection**	**Transposition near derivation**	**Transposition near bound morpheme**	**Transposition of exterior letters**
YO	7	13^*^	0	0
OR	0	7	13^*^	7^*^
BR	20^*^	20^*^	7	13^*^
MR	13^*^	20^*^	7^*^	7^*^
EL	0	20^*^	7^*^	7^*^
AD	7^*^	27^*^	7^*^	0
TL	7^*^	0	20^*^	7^*^
SK	27^*^	47^*^	13^*^	7^*^
AF	20^*^	27^*^	7^*^	0
YV	33^*^	7	13^*^	13^*^
TA	13^*^	27^*^	0	7^*^
LPD Average (*SD*)	13.4 (10.7)	19.5 (12.9)	8.5 (5.9)	6.2 (4.6)
**CONTROL GROUPS**				
12th graders	1.3(3)	2.7 (3.7)	0 (0)	0 (0)
7–8th graders	0 (0)	2.7 (4.7)	1.3 (4.2)	0.7 (2.1)
5–6th graders	0 (0)	3.3 (4.7)	0 (0)	0 (0)

* *Significantly more errors than age-matched control group (p < 0.05)*.

Each of the inflectional and derivational conditions separately yielded significantly poorer performance compared with exterior migration, *t*_(10)_ = 2.44, *p* = 0.03, *d* = 0.9, *t*_(10)_ = 3.03, *p* = 0.01, *d* = 1.4, respectively. Both inflectional and derivational conditions were each poorer than the bound function morpheme condition, a comparison that was significant for the derivational condition, *t*_(10)_ = 2.31, *p* = 0.04, *d* = 1.2, and for the initial inflectional and derivational conditions combined, *t*_(10)_ = 2.42, *p* = 0.03. As in the previous experiments, the inflectional and derivational conditions did not differ at the group level, or for any of the individuals with LPD, and neither did the bound and the exterior conditions.

## Experiment 6: comprehension of migratable words in sentences

### Materials and methods

The last experiment tested comprehension of migratable words of the various morphological structures in a more natural task in which the migratable words were incorporated into sentences. The sentences were created in a way that both the target word and the result of the transposition error are plausible in the given sentential context. The participants were requested to read each sentence silently and then to paraphrase it. We assessed whether the paraphrase reflected the target word or its transposition.

The test included 30 sentences, each with a migratable word. We compared the performance on 15 sentences with a word in which the transposition occurred adjacent to an inflectional (10 sentences) or derivational (5 sentences) morpheme, see examples (16) and (17), with 15 sentences with a word in which the transposition occurred adjacent to a bound function morpheme (18). The different conditions did not differ in frequency [*F*_(2, 27)_ = 0.16, *p* = 0.80].

(16) Migration near inflection:
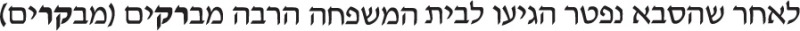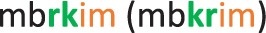 - mivrakim (mevakrim)
  After the grandpa died, there arrived to the family house many telegrams (visitors)
(17) Migration near derivation:
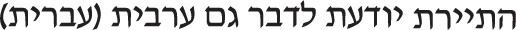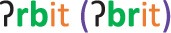 - aravit (ivrit)
  The tourist can also speak Arabic (Hebrew).
(18) Migration near a bound function morpheme:
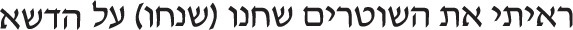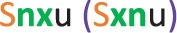 - shexanu (shenaxu)
  I saw the policemen that-parked (that-rested) on the lawn.

Sentences whose paraphrases indicated that the participant read the target word incorrectly but with an irrelevant (non-migration) error type were excluded from the analysis (16 such sentences were removed in total).

### Results

The comprehension of the migratable words in sentences, summarized in Table 12, again indicated that transpositions occurred significantly more often adjacent to inflectional and derivational morphemes than adjacent to bound function morphemes, *t*_(10)_ = 7.90, *p* < 0.001, *d* = 4.0. This pattern held also for each of the participants individually, and was significant for five of them.

**Table 12:** **Percentage migration errors in comprehension of migratable words within sentences**.

**LPD participants**	**Transposition near inflection+ derivation**	**Transposition near bound morpheme**
YO	33^*^	7^*^
OR	27^*^	7^*^
BR	21^*^	14^*^
MR	40^*^	7^*^
EL	43^*^	0
AD	20^*^	0
TL	27^*^	7^*^
SK	36^*^	0
AF	20^*^	7^*^
YV	33^*^	0
TA	40^*^	7^*^
LPD Average (*SD*)	30.9 (8.4)	5.1 (4.5)
**CONTROL GROUPS**		
12th graders	4.0 (6.0)	1.3 (3.0)
7–8th graders	5.3 (4.2)	1.5 (2.9)
5–6th graders	4.0 (4.7)	0.6 (2.0)

* *Significantly more errors than age-matched control group (p < 0.05)*.

## Letter position errors and morphology in LPD: interim summary of experiments 1–6

**Figure 1:**
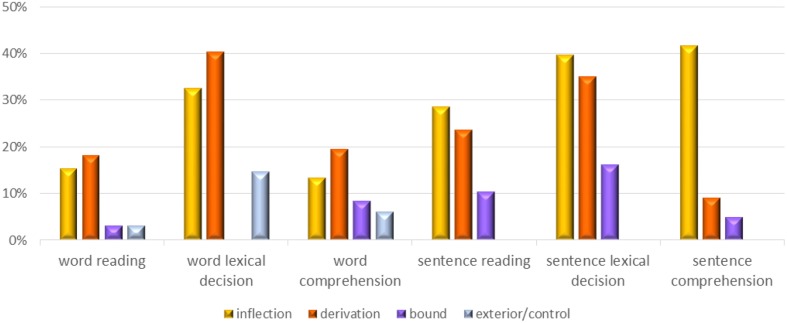
**Summary of the effect of morpheme type on migration of the root letter that is adjacent to the morpheme in the six reading experiments: Average percentage migrations**.

The pattern that the LPD participants demonstrated was consistent across the six tasks: they made very few migrations adjacent to bound function morphemes, at a rate that was similar to the low rate of exterior letter migrations, indicating they treated letters adjacent to bound function morphemes practically as exterior letters. They made significantly more migrations adjacent to inflectional and derivational morphemes. Their error rates in the various conditions in the six experiments are summarized in Figure 1.

One possible alternative explanation for the difference between letter position errors in words with bound function morphemes and words with inflectional/derivational morphemes is that bound function morphemes appear only word-initially, whereas inflectional/ derivational affixes appear both word initially and word finally (and sometimes even word-internally). However, when we compared only initial affixes, the differences between bound function affixes and inflectional/derivational affixes survived in each of the 4 experiments that included a bound function morpheme: there were significantly more transposition errors near initial inflection and derivation morphemes than near bound function morphemes, in Experiment 1, *t*_(10)_ = 10.86, *p* < 0.0001; Experiment 2, *t*_(10)_ = 3.42, *p* = 0.006; Experiment 4, *t*_(10)_ = 3.52, *p* = 0.005; and Experiment 6, *t*_(10)_ = 4.62, *p* = 0.0009.

## Similar findings from normal reading

Throughout Experiments 1–6, we had individuals with normal reading perform the same reading tasks as the LPD participants. They did not make many errors, but we were curious to see whether the few migration errors that occur in normal reading are affected by the morphological structure of the target word.

### Participants with normal reading

The participants we analyze in this section are 40 skilled readers, all Hebrew native speakers without any reading impairments according to the *TILTAN* reading screening test (Friedmann and Gvion, 2003). Twenty five of them served as age-matched controls in Experiments 1–6 and were described above in the section reporting the control participants (Section Control Group). They were tested in all 6 experiments, in the same conditions as the individuals with LPD, with stimuli presented “over the desk” for unlimited time.

Because this type of presentation yielded very few migrations in the control participants, we also added another group of 15 skilled readers, in more challenging reading conditions of limited exposure times of 300 and 100 ms. These 15 additional participants were 20–63 years old (*M* = 38.6 years, *SD* = 14.3), with 12–21 years of education (*M* = 15.3 years, *SD* = 2.4). They were tested with the word-level reading aloud, lexical decision, and comprehension tasks described in Experiments 1, 3, and 5.

### Procedure for the short exposure presentations

For the short exposure tests, the target words from Experiments 1, 3, and 5 were presented on a computer screen, for a limited time. The words for each of the three experiments (oral reading, lexical decision, comprehension) were presented in three separate blocks. Each participant saw the same 665 migratable words twice, a week apart, the words in each block were presented in a different order in the two sessions. Because most migratable words appeared in both orders in the word list (if SOFTIM appeared in the list, so did SOTFIM), and the words appeared in the list in a different order, there was no effect for remembering the words in the list. In the first session all words were presented for 300 ms (without masking). The second session, a week later, presented the same words, in a different order, for 100 ms.

In Experiment 1, the participants were requested to read each word aloud. In Experiment 3, they were requested to say, for each presented stimulus, whether it was an existing word. In Experiment 5, the participants were requested to explain each word in their own words.

### Results: migrations and morphology in normal reading

The results of the individuals with normal reading, summarized in Table 13, show that the error rate in all conditions was rather small, but still an interaction of the rates of migrations with morphological structure could be detected for normal reading as well.

**Table 13:** **Normal reading of migratable words according to the type morpheme adjacent to the transposition site**.

**Participants and task**	**Inflection**	**Derivation**	**Bound function morpheme**	**Exterior root letters**
**ORAL READING OF SINGLE WORDS**				
**Children in unlimited exposure**				
12th graders	1.2 (0.4)	1.7 (2.0)	0.3 (0.5)	0.2 (0.2)
7–8th graders	0.9 (1.1)	1.6 (1.5)	0.2 (0.4)	0.05 (0.1)
5–6th graders	1.1 (0.04)	4.0 (2.3)	0.3 (0.2)	0.1 (0.3)
**Adults in short exposure**				
Adults in 300 ms	0.6 (0.9)	2.2 (2.1)	0.3 (0.6)	0.2 (0.5)
Adults in 100 ms	1.9 (1.9)	3.5 (3.5)	0.8 (1.3)	0.1 (0.3)
**LEXICAL DECISION OF SINGLE WORDS**				
**Children in unlimited exposure**				
12th graders	3.0 (2.7)	7.0 (7.6)		4.2 (4.4)
7–8th graders	3.0 (3.5)	3.5 (5.3)		2.6 (2.8)
5–6th graders	0.5 (1.6)	4.0 (5.2)		1.1 (2.2)
**Adults in short exposure**				
Adults in 300 ms	4.3(4.9)	3.0 (3.7)		1.4 (2.4)
Adults in 100 ms	4.0 (6.2)	7.0 (6.8)		2.8 (3.4)
**COMPREHENSION OF SINGLE WORDS**				
**Children in unlimited exposure**				
12th graders	1.3(3)	2.7 (3.7)	0 (0)	0 (0)
7–8th graders	0 (0)	2.7 (4.7)	1.3 (4.2)	0.7 (2.1)
5–6th graders	0 (0)	3.3 (4.7)	0 (0)	0 (0)
**Adults in short exposure**				
Adults in 300 ms	1.8 (5.3)	3.6 (6.1)	1.3 (2.8)	0.4 (1.7)
Adults in 100 ms	6.2 (7.3)	3.6 (4.3)	2.7 (3.4)	2.2 (5.4)
**ORAL READING OF WORDS IN SENTENCES (UNLIMITED EXPOSURE)**				
12th graders	2.5 (5.6)	5.7 (7.8)	2.9 (3.9)	
7–8th graders	7.5 (8.7)	5.7 (7.4)	0.7 (2.3)	
5–6th graders	3.8 (6.0)	12.9 (8.1)	1.4 (4.5)	
**LEXICAL DECISION OF NONWORDS IN SENTENCES (UNLIMITED EXPOSURE)**				
12th graders	2.5 (5.3)	2.9 (4.5)	1.4 (3)	
7–8th graders	1.3 (4.0)	1.4 (4.5)	0.7 (2.3)	
5–6th graders	3.8 (8.4)	7.1 (12.1)	1.4 (3.0)	
**COMPREHENSION OF WORDS IN SENTENCES (UNLIMITED EXPOSURE)**				
12th graders	2.0 (4.5)	8.0 (11.0)	1.3 (3.0)	
7–8th graders	4.4 (5.3)	6.7 (10.0)	1.5 (2.9)	
5–6th graders	2.7 (4.7)	7.3 (10.1)	0.6 (2.0)	

*Average percentage errors (SD)*.

#### Adults in 100ms exposure

Not surprisingly, the condition that yielded most migrations was the shortest exposure time. In reading aloud, significantly more migrations occurred near inflection or derivation than near bound function morphemes, *t*_(14)_ = 2.54, *p* = 0.02, *t*_(14)_ = 3.85, *p* = 0.002, respectively; Similarly, significantly more migrations occurred near inflectional or derivational morphemes than in exterior letters, *t*_(14)_ = 3.71, *p* = 0.002, and *t*_(14)_ = 3.88, *p* = 0.002, respectively. In the lexical decision and the comprehension tasks a similar pattern was evinced, although only the difference between the inflection and exterior letter conditions was significant, *t*_(14)_ = 2.76, *p* = 0.02, *t*_(14)_ = 2.81, *p* = 0.02, in lexical judgment and in the comprehension task, respectively.

#### Adults in 300ms exposure

In the longer exposure condition the pattern was similar: migrations occurred more often adjacent to inflectional and derivational morphemes than adjacent to bound function morphemes and exterior letters. These differences reached significance only in the comparisons between derivation and bound function morphemes, *t*_(14)_ = 3.83, *p* = 0.002, and between inflection and exterior letters conditions, *t*_(14)_ = 3.6, *p* = 0.003.

In the lexical decision and comprehension tasks too, more migrations occurred in the letters near inflection and derivational morphemes than in letters near bound function morphemes and exterior letters, but most of these differences did not reach significance [the only significant difference was the one between the derivation and exterior conditions, *t*_(14)_ = 2.43, *p* = 0.03].

#### Children and adolescents in unlimited presentation

The unlimited presentation yielded even fewer migrations, but the same pattern persisted, although only few of the comparisons were significant, due to the ceiling effect. In *reading aloud of single words*, significant differences were found between derivational and bound function morphemes in the 5–6 and 7–8th graders [*t*_(9)_ = 6.15, *p* = 0.0002; *t*_(9)_ = 2.90, *p* = 0.02, respectively]. There were also differences that approached significance between inflection and bound function morphemes in the 7–9th graders and the 12th graders [*t*_(9)_ = 2.15, *p* = 0.057; *t*_(9)_ = 2.37, *p* = 0.08, respectively]. Significant differences were also found between inflection and exterior conditions in the 5–6th graders, 7–8th graders and the 12th graders [*t*_(9)_ = 3.03, *p* = 0.01; *t*_(9)_ = 2.55, *p* = 0.03; *t*_(9)_ = 3.76, *p* = 0.02] and between derivational and exterior letter conditions in the 5–6th graders [*t*_(9)_ = 6.0, *p* = 0.0002, and in the 7–8th graders, *t*_(9)_ = 3.26, *p* = 0.01]. No differences were found between the bound and the exterior letters conditions.

The pattern of migration errors in *reading aloud of migratable words within sentences* was similar to that manifested in single word reading. Letters adjacent to inflectional and derivational morphemes yielded more errors than letters adjacent to bound function morphemes. Given the relatively small number of migrations, only the comparisons between inflection and bound function morphemes in the 7–8th graders and derivation and bound in the 5–6 and 7–8th graders reached significance, *t*_(9)_ = 2.5, *p* = 0.003; *t*_(9)_ = 3.2, *p* = 0.001; *t*_(9)_ = 2.33, *p* = 0.04, respectively.

The same tendency was found in *lexical decision of single words*, where only the difference between derivational and exterior letter conditions in the 5–6th graders reached significance, *t*_(9)_ = 2.22, *p* = 0.05. In the *Lexical decision of nonwords in sentences* the same tendency emerged, without significant differences; In *comprehension of single words* significant differences were found only in the performance of the 5–6th graders between derivational and bound function morphemes and between derivational and exterior letters [both comparisons yielded *t*_(9)_ = 2.24, *p* = 0.05]. Finally, in *comprehension of words within sentences*, the 7–8th graders made significantly more errors in the inflectional and derivational condition compared to the bound condition, *t*_(9)_ = 1, *p* = 0.02.

## Discussion

This study examined the nature of early morphological decomposition in reading via testing letter position errors that individuals with LPD make in words of various morphological structures. The study was based on the well-established finding that in LPD almost only middle letters migrate whereas exterior letters are less prone to errors. We used this fact to ask whether morphological decomposition occurs prior to letter position encoding: we reasoned that if words are decomposed to their roots and morphological affixes, then letters that used to be internal in the visually perceived complex word become exterior following decomposition (such as in the case of the English word *signs*, where the letter *n* is internal in the complex word, but is exterior in the base *sign*). Thus, if such word-internal base-exterior letters do not migrate, this can indicate that morphological decomposition affects letter position encoding, and hence, precedes it. We compared three types of morphological affixes: inflectional, derivational, and bound function morphemes.

### The ordering of morphological analysis and letter position encoding

The assessment of the effect of morphology on letter position errors in LPD indicated that morphological decomposition follows letter position encoding for inflectional and derivational morphology. This makes sense: it is hard to imagine how morphological analysis of a morphologically complex word can proceed before the order of the letters is encoded (after all, -ment is a suffix, but -nemt is not). We reached this conclusion on the basis of the finding that letter position errors occurred in the root letters adjacent to inflectional and derivational affixes even when morphological decomposition would make these letters exterior and hence less liable to migrations. Namely, letter position errors occurred prior to the analysis of the inflection and derivation in morphologically complex words.

The results also clearly indicated that letter position errors are sensitive to the morphological structure of the target word: whereas the participants made migration errors on the letters that were adjacent to inflectional and derivational morphemes, treating them as middle letters, they did not make almost any migration errors on root-exterior letters that were adjacent to a bound function word (namely, when encountered with a letter string composed of a bound function morpheme and a word, parallel to *the-art* in English, they almost never made exterior errors in the word base that would lead to reading it as “the-rat”).

We suggest that these results can be explained if one distinguishes between morphological *analysis* and morphological *decomposition*. The results are consistent with the following model: the first stages of word reading involve letter identification and letter position encoding. Then, an early, prelexical, morphological analysis takes place, whereby the morphological structure of the word, including inflection, derivation, and bound function morphemes, is analyzed. This analysis is structural in nature, non-lexical, and it relies on knowledge of existing inflectional and derivational templates.

This analysis is enough for morphologically complex words that include inflectional and derivational morphology to access the next stages of reading: the orthographic input lexicon and the sublexical route. Letter position encoding occurs prior to this morphological analysis and hence letter errors affect letters of the root even if they are adjacent to inflectional or derivational morphemes. We assume that in inflectional and derivational morphemes, the system encodes letter position for the whole complex word, and then during the morphological analysis, when the three letters of the root are extracted, they receive their letter position within the root directly as part of the analysis: if the word is *XKRh* (she-researched), where the root is XKR and the *h* is the feminine singular affix, it is enough to encode the position of the letters to know that K is the second letter of the root. The same with derivational prefixes like *m* in the word *mXKR* (research). In this case, again, the morphological analyser that analyses the *m* as the derivational prefix and XKR as the root can already assign the letter position of the root letters and hence, again, K will be encoded as the second letter of the root. A letter position encoding error would therefore affect the position of all middle letters in the morphologically complex word, including the letters on the verge of the inflectional and derivational morphemes.

**Figure 2:**
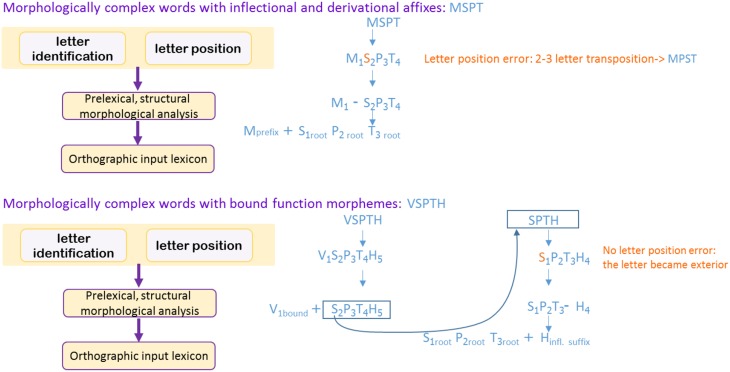
**The model of morphological analysis and decomposition in inflectionally/derivationally complex words vs. words with bound function morphemes**.

The story is different when this early morphological analysis detects that the letter string cannot be analyzed as including a root and inflectional and derivational morphemes, as is the case in words with bound function morphemes like *the-art* (which, in English and many other languages visually appear as two words). In this case, the string is decomposed into the two constituents (the function word *the* and the word *art*), and then letter position encoding should take place again on the two constituents (or at least—on the base constituent, because the position of the bound function morpheme is already encoded as first). This might be because once decomposed, the letter positions of the base word (which could be morphologically complex in itself) changed and should be re-coded (a letter that was second prior to decomposition now becomes the first letter in the base)^7^. When this happens, and letter position encoding is applied to the decomposed words, letter position errors again occur only in middle positions of the constituent words, and hence the exterior letters of the constituent words do not migrate^8^. We summarize and exemplify our proposed model in Figure 2 (and see Appendix C for a transcribed example and a parallel example in English).

Of interest is also the finding that there were practically no transpositions across a function morpheme boundary: the letter of the function morpheme almost never transposed with the letters of the root, suggesting another corroboration for the conclusion that morphological decomposition of bound morphemes occurs prior to letter position encoding.

The results showing the morphological effects on letter position errors were consistent across the 6 experiments, on words presented in isolation and within sentences, and were evinced both in the reading of the participants with LPD and in the reading of the skilled readers, who made much fewer errors, but with the same patterns.

### Morphological analysis is prelexical

Our results also suggest some further insights as to the nature and locus in the reading process in which morphological analysis occurs: they suggest, like many previous studies, including studies on morphological analysis in peripheral dyslexias, that the morphological analysis does not rely on lexical considerations but rather on a structural analysis of the words.

This conclusion is supported by three findings in the current study: firstly, LPD is a deficit at the letter position encoding function in the early, pre-lexical stage of orthographic-visual analysis. The fact that morphological structure affects letter position errors, at least in the case of bound function morphemes, suggests that morphological analysis occurs in this early stage of orthographic-visual analysis.

Secondly, there were words that started with a bound function morpheme but could structurally be analyzed as starting with a verbal derivational affix, although the root does not exist with this derivational affix (see Appendix B). Such words were analyzed as starting with a derivational affix, as indicated by the higher rate of migrations adjacent to their first letter. The fact that the analysis created a non-existing word indicates that the analysis was structurally, rather than lexically driven (in line with previous studies such as Longtin et al., 2003; Rastle et al., 2004; Longtin and Meunier, 2005; Rastle and Davis, 2008; Reznick and Friedmann, 2009; Beyersmann et al., 2011; Crepaldi et al., 2014), supporting the conclusion that morphological analysis takes place in an early, pre-lexical stage.

This conclusion of pre-lexical morphological analysis is also supported by the finding that nonwords showed exactly the same morphological effect as words: Experiments 3 and 4 showed that even in morphologically complex nonwords, in which both the whole nonword and its root did not exist, there were much fewer migration errors adjacent to a bound function morpheme than adjacent to inflectional and derivational morphemes^9^.

Such pre-lexical, structurally-based analysis takes place both when reading a whole sentences and when morphologically complex words are presented in isolation.

### A note on accounts for developmental dyslexia

Given that 11 of the participants had developmental LPD, the results also shed light on the source of developmental LPD, and, more specifically, shed light on what cannot be the source of developmental LPD.

Firstly, as in many other cases of developmental LPD (Friedmann and Rahamim, 2007), all of the participants had perfect repetition of migratable words, indicating good phonological production, and all of them had normal picture naming, indicating good lexical retrieval processes. This demonstrates that neither phonological impairment nor lexical impairment can account for their dyslexia (cf. Castles and Coltheart, 2004; Castles and Friedmann, 2014). Rather, they showed a selective deficit in letter position encoding.

Furthermore, some studies ascribe developmental dyslexia to impaired morphology (Shu et al., 2006). Here we actually saw the exact opposite: the preserved morphological ability of our participants with LPD modulated dyslexic errors and protected the letter position dyslexics from making errors in one of the conditions. Additionally, in their reading aloud and in their word repetition they did not make morphological errors: they did not substitute or omit morphological affixes. Thus, developmental dyslexia, or at least developmental LPD, does not originate in a morphological impairment.

Finally, and this applies to both developmental and acquired LPD, some accounts for letter migrations provide visually-based explanations for the relative immunity of exterior letters to letter migration. The current results suggest that this cannot be the whole story, because stem-exterior letters may be immune to migrations even when they visually appear word-interiorly. We saw that first letters of the root, when appearing right after a bound function morpheme, very rarely migrate, and their migration rate is comparable to that of first letters of the root that are also visually exterior. This suggests that morphological-orthographic processing also contribute to the relative immunity of exterior letters.

Therefore, the results of the current study show that developmental LPD does not stem from a phonological, lexical, morphological, or visual impairment. These results thus are also inconsistent with general claims that do not distinguish between different types of developmental dyslexia, which suggest that one of these factors is the source of developmental dyslexia in general.

### Conflict of interest statement

The authors declare that the research was conducted in the absence of any commercial or financial relationships that could be construed as a potential conflict of interest.

## Footnotes

^1^Whereas letter identification and letter position encoding have to occur in the first stage of orthographic-visual analysis, letter-to-word binding may occur slightly later, in the graphemic input buffer, which holds the products of letter identification and position stages, a stage that can hold more than a single written word at a time.

^2^Most of these bound function morphemes have a full-word counterpart that appears as a stand-alone function word. Talmy Givón (1971) made the famous claim “Today's morphology is yesterday's syntax”—according to which in many languages bound morphemes arise historically from free lexical morphemes. The same is true for Hebrew bound function words. Historically, most of these morphemes started out as the full independent form and then their phonologically reduced form emerged, written as a bound prefix. For example, “min,” from, became “mi-,” “kemo,” like, became “ke-,” and “el,” to, became the attached “le-” (Hardy, 2014 and cf., Pat-El, 2012, for a discussion of the relation between the full independent relativizer “asher” and the bound clitic “she-”). Given that most full-forms still exist alongside the bound morphemes, this may contribute to the perception of such bound function morphemes by Hebrew speakers as separate words.

^3^In Hebrew, due to the under-specification of vowels in the orthography, and to the fact that there are 9 letters that have an ambiguous conversion to phonemes, 13 homophonic letters, and lexical stress that is not marked in the orthography, there are actually no regular words. Therefore, all words in the screening tests were irregular, but for the detection and identification of surface dyslexia, we used the two types of words that are most sensitive to surface dyslexia: potentiophones –words whose reading via grapheme-to-phoneme conversion creates another existing word, like *now*, which can be read via grapheme-to-phoneme conversion as “know” (Friedmann and Lukov, 2008), and words that are parallel to irregular words in English—words with silent letters or with a letter that can be converted via the sublexical route into two or more different phonemes, and in the target words the letter is converted to the less-frequent conversion phoneme.

^4^As the screening test reading (Table 2) indicates, six of the participants with developmental LPD also made surface-dyslexia-like errors, resulting from reading via grapheme-to-phoneme conversion instead of via the lexical route, in a higher rate than expected for their age. These errors do not necessarily mean that these participants have surface dyslexia on top of their LPD, but could rather stem from their insufficient exposure to reading because of the reading difficulties, which results in insufficient entries in the orthographic input lexicon, forcing them to read through the sublexical route. It might also be that given the pre-lexical deficit in the orthographic-visual analyzer, the representations in their orthographic input lexicon are abnormal.

^5^We selected this exterior control condition because we were interested in whether the migrations adjacent to a morpheme behave like exterior migrations. A middle letter migration control condition, which involves migration of middle letters within the root and not adjacent to morphemes, is impossible in Hebrew because Hebrew words are based on 3-letter roots. Interior letter migrations require at least 4 letters, but 4 letter words inevitably include affixes. Therefore, middle letter control items that involve migration of two letters of the root and do not include affixes are impossible (or are limited to loan words that do not have the Semitic morphological structure).

^6^We could not compare these migrations to migrations in the middle of the root because Hebrew roots are generally 3-letter roots, so there is no way for a migration to involve two letters in the middle of the root, and hence every migration of middle letters is on the edge of a suffix.

^7^An important distinction, which might underlie the reason why words that appear with a bound function morpheme need to be decomposed and return for re-encoding of letter positions, is the distinction between words and roots. Whereas derivational and inflectional morphemes in Hebrew (and in Semitic languages in general) appear with a root, bound function morphemes appear with a word. This word, in turn, may be morphologically complex in itself. Morphological analysis of morphologically complex words that are composed of a root and inflectional and derivational affixes is enough to allow access to the lexicon, because the identification of the derivational template and inflections provides information about the slots of the three consonants of the root and their order. Such analysis is impossible when a morphologically complex word appears bound to a bound function word. In this case, it seems that to apply morphological analysis and identify letter positions within the root, the word needs to be decomposed and stripped off the bound morpheme, and then fed to the process again, for re-encoding of letter position, followed by the iteration of the morphological analysis stage.

^8^Interestingly, Taft and Nillsen (2013) did not find any difference between priming of prefixed words in which stem-initial letters transposed and those in which stem-interior letters transposed (as was the case in our study with inflectional and derivational prefixes). They explained this finding in that although morphological decomposition is prelexical, the initial letters are less prone to transpositions due to their perceptual salience. The results of our study indicate that there actually can exist cases in which the morphological structure is decomposed in a way that stem-initial letters that are not perceptually initial are resistant to transpositions, hence supporting the idea of early morphological decomposition, but suggesting that the effect of morphology on letter position encoding differs between different morpheme types.

^9^Whereas words like the-art in Hebrew appear orthographically as one word but can be decomposed structurally based on knowledge of morphology and without any contribution of lexical knowledge, word-word compounds that occur in some other languages may require a different treatment. In languages like German and Italian, compounds may be created from two or more words combined (Kirschfruchtfaft, tostapane, see a recent special issue on compounds, Semenza and Luzzatti, 2014). In these cases, the decomposition of compounds into their constituting words is probably lexically-based rather than purely structural.

## Appendix A

**Table A1:** **Examples for Hebrew words with the root , SPR, with inflectional, derivational, and bound function morphemes. The root meanings relate to stories, numbers, and hair-cutting**.

**Hebrew**	**Transliteration**	**Transcription**	**Translation**
**INFLECTION**			
		safarti	counted-past-1sg
		safart, safarta	counted-past-2sg-fem counted-2sg-mas
		safra, sifra	counted-past-3sg-fem, her book (also derivation: digit)
		safarnu	counted-past-1pl
		safartem	counted-past-2pl-mas
		safarten	counted-past-2pl-fem
		safru	count-past-3pl
		espor	count-fut-1sg
		tesapri, tisperi	cut-hair-fut-2sg-fem, tell-fut-2sg-fem, count-fut-2sg-fem
		yispor	count-fut-3sg-mas
		nispor	count-fut-1pl
		tisperu, tesapru	count-fut-2pl, cut-hair-fut-2pl, tell-fut-2pl
		yisperu, yesapru	count-fut-3pl, cut-hair-fut-3pl, tell-fut-3pl
		sofer	counts-mas (also derivational: author-mas)
		soferet	counts-fem (author-fem)
		sofrim	count-pres-pl-mas (author-pl-mas)
		sofrot	count-pres-pl-fem (author-pl-fem)
		sfarim, saparim	books, barbers
		sifri, sfarai, sapri	my-book, my books, tell-imperative-fem-sg
		sifrex, sifrexa	your-fem-book, your-mas-book
**DERIVATION**			
		safran	librarian-mas
		sifriya	library
		sifrut, sfarot, saparut	literature, digits, hairdressing
		histaper	cut-hair-refl (got a haircut)
		siper	told, cut-hair
		nispar (nesaper)	was-counted (can also be inflection: tell-fut-1pl)
		saparit (sifriyat)	hairdresser-fem (library-of)
		sipur	story
		siporet	fiction
		mispar (mesaper)	number (can also be inflection: tells-3sg-mas)
		misperet (mesaperet)	scissor-kick (can also be inflection: tells-3sg-fem)
		sifron	booklet
		mispara	barbershop
		misparayim	scissors

		histaparnu	we-got-a-haircut (cut-hair-refl-1pl)
		sipartem	told-past-2pl, cut hair-past-2pl
		nisperu	were-counted
		mistaprot	getting-a-haircut-pres-pl-fem
		misparim	numbers
		safraniyot	librarian-fem-pl
**BOUND FUNCTION MORPHEMES**			
		hasefer	the-book
		vesefer	and-book
		shesafar	that-counted, that-a-book
		lasefer, lesefer (lesaper)	to-the-book, to-a-book (also inflectional: to tell)
		misefer (mispar)	From-a-book (also derivational: number)
		basefer, besefer	in-a-book, in-the-book
		kesefer	as-a-book

*The root appears in purple, inflectional morphology in orange, derivational in blue, bound function morphemes in cherry red*.

## Appendix B

### What ambiguous letters tell US about morphological decomposition?

We saw that bound function morphemes behave differently from other morphemes in their effect on letter position errors. As a next step we asked whether it is the letter itself that is identified as a “function letter” by the morphological mechanisms in the orthographic-visual analyzer, or whether a first-pass analysis of the whole word is done. For this aim, we made two additional analyses that took advantage of the fact that some Hebrew letters can represent both bound function morphemes and inflectional or derivational morphemes, and some can be both bound function morphemes and root letters.

#### Analysis of letters that can function as bound function morphemes or as inflectional/derivational morphemes

The letters  (corresponding to the function words ha-,me-,le-), when appearing as the first letter of the word, form the bound function morphemes “the,” “from,” and “for/to” respectively, but they can also function as inflectional and derivational morphemes when they appear as the first letter of the word, before the root. When they function as inflectional/derivational morpheme, they are part of the morphological structure of the word, which often includes other letters in the middle or end of the word, that form part of its morphological structure.

We examined the rate of transposition errors near the three ambiguous letters when they functioned as bound function morphemes, and compared them to words in which they functioned as inflectional or derivational morphemes. We also compared the rate of transpositions near these letters when they functioned as bound function morphemes compared to the rate of transpositions near non-ambiguous bound function morphemes () (be-,ke-,ve-,she-), and the rate of transpositions near these letters when they functioned as inflectional and derivational morphemes compared to transpositions near non-ambiguous inflectional or derivational morphemes () (a,i,t,n).

This analysis revealed that when the first letter functioned as an inflectional or a derivational morpheme, and was part of a derivational/inflectional structure, the LPD participants made three times more transpositions (13.2%) near it than when it functioned as a bound function morpheme (4%), a difference that was significant, χ^2^ = 22.13, *p* < 0.0001. The letters that can function both as bound and as inflectional/derivational morphemes showed similar transposition rates to the non-ambiguous bound function letters (3%) when they functioned as bound (4%) (χ^2^ = 0.29, *p* = 0.59), and similar to the non-ambiguous inflectional/derivational letters (12.2%) when they functioned as inflectional/derivational (13.2%), (χ^2^ = 0.23, *p* = 0.63). These findings suggest that the difference is not only based on the identification of the first letter as one of a list of “function letters” but is rather based on a wider morphological analysis that looks at the structure of the whole word and its properties.

#### Analysis of letters that can function as bound function morphemes or as root letters

All letters that are part of morphological affixes can also function as root letters. We analyzed the letters  and  (corresponding to the function words *be*-, in, and *she*-, that), the two letters for which we had enough instances in which they appeared both as a bound function morphemes and as first letters of the root, followed by at least 3 consonant letters.

We compared the rate of transposition errors near the two ambiguous letters when they functioned as bound function morphemes, and when they functioned as the first letter of the root. In this analysis we only included words in which a structural-morphological analysis can identify the role of the first letter: we therefore selected words with at least 4 letters in which the first letter was the ambiguous , the 2nd and 3rd letters were consonants and their transposition created another existing word (17 words in which  functioned as a root letter and 21 words in which they functioned as bound function morphemes).

For example,  (BXRTM, baxartem, you-pl-chose), starts with the ambiguous letter B, which functions in this word as a root letter. A structural analysis of this string would suggest that the first letter is a root letter because the last two letters form a suffix, so the three consonants of the root need to include the first letter. In contrast, in the word , BSɁR, ba-sha'ar, in-the-gate, the letter B functions as a bound function morpheme. In this word, there are three consonant letters after the first letter, none of which could be affixes, indicating that the first letter is a prefix - a bound function morpheme.

The results, again, indicated that a full morphological analysis of the word is done, and not only identification of the first letter as one of a list of bound function morpheme letters. Twice as many migrations occurred in the (2nd and 3rd) letters that are adjacent to the ambiguous first letter when the first letter served as a root letter than when it served as a bound function morpheme. An average of 1.7 migrations occurred (for the 11 developmental LPD participants combined) when the first letter  was a root letter, very similar to the rate of errors in words in which the first letter was unambiguously a root letter (1.6), and only 0.7 migrations when it was a bound function morpheme.

This suggests that the morphological analysis takes into account the whole structure of the word, the existence of three consonant that could function as the root and the existence of other letters that can function as derivational or inflectional morphemes.

Finally, a further interesting finding relates to words that structurally could be analyzed as words (verbs) in a derivational template, but lexical knowledge actually indicates that this is a composition of a bound function morpheme and an existing word, whereas the derivational form is a non-word. (For example, the word with the bound determiner , the-prisoner, is cast in the derivational template for causative verbs, , but such verb does not exist in this template). Thus, had the analysis been guided by lexical considerations, such words would be analyzed as a bound function morpheme+word, and lead to reduced migration rate. But in fact, they were structurally analyzed as one derivationally complex word, as signaled by the fact that migrations occurred after the first letter. There were 45% migration errors on this word, exactly like the average rate of migrations in real verbs in this template, whereas there were only 3.8% errors in the rest of the words in the list that started with the bound article and did not conform to an existing verbal template.

These analyses indicate that the morphological parsing takes into account possible morphological templates and affixes, and analyzes the whole word. If three consonant letters are followed by letter(s) that are recognized to be a suffix, the first letter is analyzed as a root letter. If, however, a consonant letter that can be a bound function morpheme is followed by a morphological structure that includes three root letters in a known template, it is analyzed as a function morpheme, and hence is subject to decomposition and stripping off from the following word. Notice that such analysis can occur early, structurally, and without any access to the lexicon, but rather be based solely on knowledge of the existing templates and affixes, their position within the word, and the demand for three root consonant letters.

## Appendix C

The differential information processing of derivationally complex word and a word with bound function word.

The Hebrew examples: a. , MSPT (sentence or trial), derived from the derivational template MXXX and the root SPT. b. , VSPTH (and-judged-fem), in which the bound function morpheme “and,” V- is attached to the morphologically complex word she-judged SPTH, which is itself combined of the root SPT and the feminine singular past inflection -H.

A parallel example in English would be the following, however, notice that it cannot reflect the whole story of Semitic languages like Hebrew, because there is no notion of the letters of the root and their order, neither is there a single letter that functions as a bound function prefix:
